# Hospital Pharmacists as Examiners

**Published:** 1920-11-13

**Authors:** 


### Hospital Pharmacists as Examiners.

Amongst those persons appointed to examine candidates in the examinations for the statutory qualification of pharmacist we note the names of five prominent hospital pharmacists—viz., Mr. C. H. Hampshire, B.Sc., Chief Pharmacist, University College Hospital; Mr. F. A. Hocking, B.Sc., Chief Pharmacist, London Hospital; Mr. J. A. Jennings, Ph.C., Chief Pharmacist, St. Thomas's Hospital; Mr. J. W. Peck, Ph.C., Chief Pharmacist, Great Ormond Street Hospital (Children's); Mr. A. L. Taylor, Ph.C., Chief Pharmacist, Bristol Royal Infirmary. A compulsory curriculum, has now been instituted for the qualification of pharmacist, and the "final" examination is now divided into two parts.

